# Lower limb joint angle variability and dimensionality are different in stairmill climbing and treadmill walking

**DOI:** 10.1098/rsos.180996

**Published:** 2018-12-12

**Authors:** P. C. Raffalt, S. Vallabhajosula, J. J. Renz, M. Mukherjee, N. Stergiou

**Affiliations:** 1 Julius Wolff Institute for Biomechanics and Musculoskeletal Regeneration, Charité – Universitätsmedizin Berlin, Berlin, Germany; 2 Department of Biomedical Sciences, University of Copenhagen, Copenhagen, Denmark; 3 Department of Physical Therapy Education, School of Health Sciences, Elon University, Elon, NC, USA; 4 Department of Biomechanics, College of Education, University of Nebraska Medical Center, Omaha, NE, USA; 5 Department of Environmental Agricultural and Occupational Health, College of Public Health, University of Nebraska Medical Center, Omaha, NE, USA

**Keywords:** biomechanics, gait, variability, nonlinear dynamics, kinematics, stairs

## Abstract

The present study tested if the quadratic relationship which exists between stepping frequency and gait dynamics in walking can be generalized to stairmill climbing. To accomplish this, we investigated the joint angle dynamics and variability during continuous stairmill climbing at stepping frequencies both above and below the preferred stepping frequency (PSF). Nine subjects performed stairmill climbing at 80, 90, 100, 110 and 120% PSF and treadmill walking at preferred walking speed during which sagittal hip, knee and ankle angles were extracted. Joint angle dynamics were quantified by the largest Lyapunov exponent (LyE) and correlation dimension (CoD). Joint angle variability was estimated by the mean ensemble standard deviation (meanSD). MeanSD and CoD for all joints were significantly higher during stairmill climbing but there were no task differences in LyE. Changes in stepping frequency had only limited effect on joint angle variability and did not affect joint angle dynamics. Thus, we concluded that the quadratic relationship between stepping frequency and gait dynamics observed in walking is not present in stairmill climbing based on the investigated parameters.

## 1. Introduction

The complex movement of human walking has been modelled as an inverted pendulum where the centre of mass (CoM) travels in a series of arcs during each single support phase while the stance leg is kept relatively straight [1,2]. Based on this model, the preferred stepping frequency (PSF) of healthy individuals has been estimated by computing the resonant frequency of a force-driven harmonic oscillator [3]. The PSF has been linked to decreased energy expenditure [4,5], decreased lower limb muscle activation [6] and decreased positive ankle joint work when compared with higher and lower stepping frequencies [7]. These results and others [8,9] have led to a well-established conclusion in the literature, that there is a quadratic relationship between walking speed and energy expenditure.

Furthermore, gait dynamics also seem to exhibit similar relationships with stepping frequency. Specifically, walking at PSF coincides with a minimum rate of trajectory divergence of the knee joint angle (measured by the largest Lyapunov exponent) and minimum amount of variability of the movement trajectories in comparison to stepping frequencies above and below PSF [10]. This relationship has also been observed for a range of walking speeds above and below the self-selected preferred walking speed (PWS) [11]. Other studies have also observed a quadratic relationship between walking speed and the temporal dynamics of stride interval characteristics [12,13]. Thus, it seems to be both mechanically and energetically disadvantageous to force the stepping frequency during walking and the walking speed above or below what is freely chosen.

The above data can also provide support for dynamical systems theory which suggests that a behavioral solution to a movement problem can arise from the synergistic organization of the neuromuscular system incorporating the constraints from environmental, biomechanical, biological and task-related factors [14,15]. These behavioral solutions can be characterized by the study of the coordination patterns (e.g. in terms of rate of trajectory divergence, dimensionality and regularity) and the quantification of their variability [15]. Therefore the above-presented data would suggest that an optimal movement solution is exhibited during walking at PSF and PWS [10–13]. However, even though such observations and inferences can be made for walking it is unknown if this is also the case for stairmill climbing which shares obvious similarities with walking.

Recently, we observed that the temporal dynamics of stride interval characteristics during continuous stairmill climbing at PSF differed from that of treadmill walking and that these dynamics did not change with increase in stepping frequency during stairmill climbing [16]. While these observations do not support the existence of an optimal movement solution at PSF for continuous stairmill climbing, they were limited to stride-to-stride characteristics and stepping frequencies above PSF. Furthermore, it could be questioned whether stride-to-stride gait dynamics captures the inherent gait characteristics or joint angle dynamics would be a better choice. Therefore, the logical next step is to investigate joint angle dynamics and joint angle variability at a range of stepping frequencies during continuous stairmill climbing.

Therefore, the purpose of the present study was to investigate joint angle dynamics and joint angle variability during continuous stairmill climbing at stepping frequencies both above and below PSF. We used continuous stairmill climbing as it constitutes a familiar locomotor task which can be performed at a range of stepping frequencies. We hypothesized that the dynamics of the oscillatory leg movements would not differ between stairmill climbing and treadmill walking. Joint angle variability was assessed by the average variation of the sagittal joint angle movements and movement dynamics was assessed by the largest Lyapunov exponent (LyE) and the Correlation Dimension (CoD) of the joint angle trajectories. The LyE and CoD quantify different characteristics of the dynamics of a time series [17,18] and have previously been successfully used to describe the complex behaviour of human movement [11,19–21]. If stairmill climbing at PSF shares the same principal dynamics for the oscillatory leg motions with treadmill walking at PSF, increase or decrease in stepping frequency would alter the joint angle dynamics and variability. We hypothesized that stepping frequencies above and below PSF would increase joint angle variability and change the joint angle dynamics through an increase in the LyE and the CoD.

## 2. Method

The present study included analysis of data collected during the experimental part of a previous study [16]. The previous and present study share the same subject and experimental equipment (motion capture system, treadmill and stairmill), but the previous study included only a part of the protocol applied in the present study. Specifically, the previous study investigated walking at PWS on the treadmill and stairmill climbing at 100%, 110% and 120% PSF. Furthermore, the previous study investigated the dynamics of stride characteristics (stride time, stride length and stride speed) using detrended fluctuation analysis and sample entropy during the two tasks. The present study has an extended experimental protocol and includes analyses of unpublished data.

### 2.1. Participants

Nine healthy participants (five females and four males) with a mean (s.d.) age of 25.2 (4.9) years, body height of 1.71 (0.10) m and body mass of 69.1 (13.8) kg were recruited for the present study. Before inclusion in the study, the following criteria should be met: (1) aged between 19 and 35 years, (2) familiarity on both tasks (stairmill climbing and treadmill walking) and (3) ability to provide informed consent. The included participants had no known sensory, neuromuscular, skeletal or cardiovascular disorders. All participants gave their informed written consent after receiving information on the experimental condition and study purpose. The study was approved by the Institutional Review Board of the University of Nebraska Medical Center and the study was carried out in accordance with the approved guidelines.

### 2.2. Experimental set-up and procedure

Kinematics were collected during stairmill climbing and treadmill walking at 60 Hz using a 12 high-speed camera system (Motion Analysis Corp., Santa Rosa, CA) and 27 retro-reflective markers placed bilaterally on: 1) anterior superior iliac spines, 2) posterior superior iliac spines, 3) greater trochanters, 4) mid-lateral thighs, 5) lower front thighs, 6) lateral knees, 7) tibial tubercles, 8) lower lateral shanks, 9) lateral ankles, 10) top of the second metatarsophalangeal joints, 11) posterior heels, 12) lateral fifth MTPs and 13) lateral calcanei. An additional single marker was placed on the sacrum.

**Figure 1.**
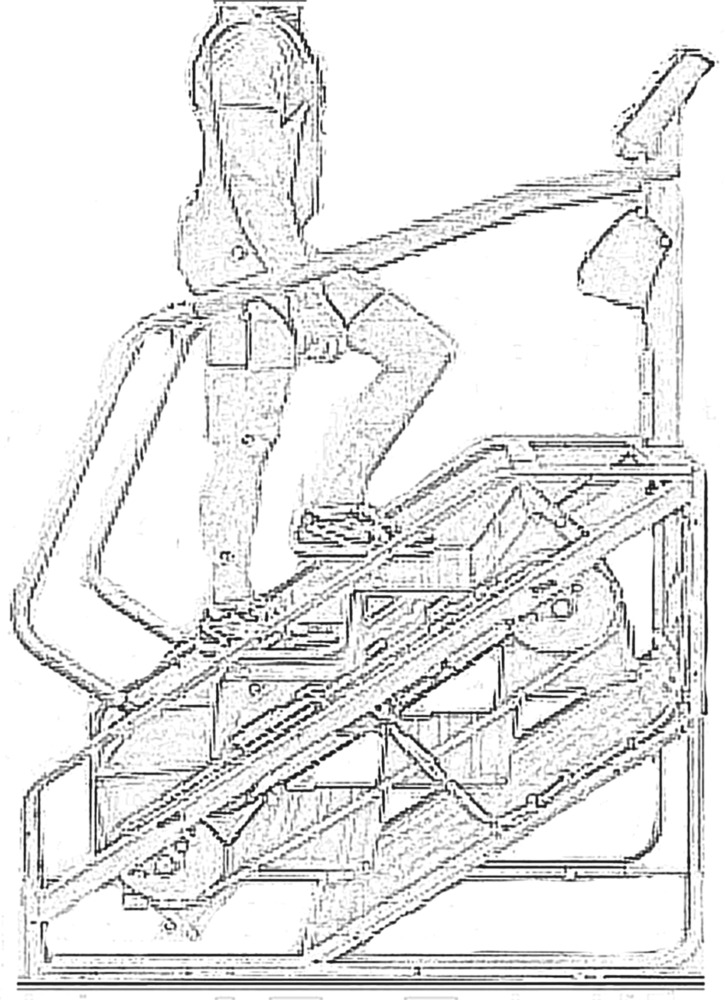
Schematic picture of the stairmill experimental set-up.

Continuous stairmill climbing was performed on a SC916 motorized stairmill (StairMaster, Fitness Direct, San Diego, CA; figure 1), while treadmill walking was performed on a T280S flat motorized treadmill (Bodyguard Fitness, QC, Canada). The participants were instructed to climb on the stairmill without the use of the handrails at their PSF. The selected stepping rate was confirmed by increasing the stepping rate until the participants reported discomfort with further increase. The stepping rate was then decreased until the participants reported it was below PSF. This procedure was repeated until a comfortable PSF was reported by the participants. The numerical value of the stepping rate was hidden from the participants at all times. A 1-min familiarization trial at PSF was performed by each participant followed by at least 2 min rest before data collection trials were initiated.

The participants completed five 3 min trials at different stepping rates (80, 90, 100, 110 and 120% of PSF) in randomized order. Between each trial, a resting period of at least 3 min was provided. Following the final stairmill trial and after sufficient rest, the PWS during treadmill walking was determined by repeatedly increasing and decreasing the walking speed above and below what was reported by the participants as comfortable. Finally, following sufficient rest, participants completed a 5 min waking trial at their PWS. The PWS was on average 1.19 (0.24) m s^−1^.

### 2.3. Data analysis

All analysis of the unfiltered marker position data was processed using Matlab (Mathworks, Inc., Natrick, MA). On both the stairmill and the treadmill, toe-off events were identified from abrupt change in the anterior–posterior displacements of the toe marker indicating the change from a backward to a forward motion during the contact phase. Stride phase was calculated as the time interval between two consecutive toe-off events on the right leg and 50 consecutive strides were extracted. Stride frequency during the treadmill walking trial and the stairmill climbing trials was calculated as the inverse of stride time and averaged across all 50 strides. Hip, knee and ankle joint angles on the right leg were calculated using a modified Helen Hayes marker set-up algorithm [22].

The process of quantifying joint angle variability included the following steps: (1) time-normalize the joint angles to 100% of each stride, (2) calculate the standard deviation across all strides at each time point and (3) average the standard deviation across the 50 time-normalized stride phases (meanSD) [23,24]. Thus, meanSD quantifies the total amount of spatial variation in the joint angle trajectory relative to the stride phase.

The method applied to quantify movement dynamics has been described in detail elsewhere [11,25,26] but is briefly described below as well. The LyE estimates the rate of divergence or convergence of trajectories of a time series in its state space [18]. High LyE values are observed in time series with a more random spatio-temporal structure and low LyE values are observed in time series with a more periodic spatio-temporal structure. The CoD quantifies the organization of data points in state space and approximates the fractal dimension of the region in the state space occupied by the dynamical system in question [17,19,27]. Time series with high fractal dimensionality and greater flexibility in the state space organization are characterized by high CoD [11]. The present study used the algorithm from Wolf *et al*. [18] for LyE calculation and the algorithm from Grassberger & Procaccia [17] for CoD calculation. Before calculating LyE and CoD, the time series in question was reconstructed in state space using the method of delay embedding [28–30]. The time delay (Tau) and embedding dimension (EmD) were calculated for each joint angle time series during each of the five stairmill trials and the walking trial using the Average Mutual Information and False Nearest Neighbor algorithms, respectively [21,31]. The group mean and standard deviation of Tau and EmD for each walking condition and each joint are presented in table 1. To best represent all investigated time series for the walking trials, a Tau of 15 and an EmD of 5 were chosen for all state space reconstructions. Correspondingly, a Tau of 32 and an EmD of 7 were chosen for all stairmill climbing state space reconstructions. To verify that the number of included strides in the analysis did not affect the results, additional analyses were completed. LyE and CoD calculated from time series including a range of strides of 10–50 for stairmill climbing and of 10–150 for treadmill walking was investigated. The results of these analyses are summarized in the Results paragraph and presented in detail in the electronic supplementary material.

**Table 1.** Group mean and SD for Tau and EmD for hip, knee and ankle angles during treadmill walking at PWS and stairmill climbing at 80, 90, 100, 110 and 120% of PSF and mean and SD for all stairmill conditions.

	hip		knee		ankle	
	Tau	EmD	Tau	EmD	Tau	EmD
treadmill						
PWS	17.4 ± 1.6	5.0 ± 0.5	15.5 ± 4.6	5.3 ± 0.7	10.7 ± 5.1	5.3 ± 0.5
stairmill						
80% PSF	41.1 ± 8.6	5.9 ± 0.6	41.7 ± 11.0	7.2 ± 0.8	28.4 ± 6.0	7.0 ± 1.2
90% PSF	37.3 ± 7.2	5.9 ± 0.3	38.1 ± 11.0	7.3 ± 0.9	25.7 ± 4.5	6.8 ± 1.5
100% PSF	35.8 ± 5.2	6.1 ± 0.6	37.0 ± 6.7	6.7 ± 0.7	24.8 ± 3.3	7.0 ± 1.0
110% PSF	31.4 ± 5.2	5.9 ± 0.6	33.2 ± 5.7	7.0 ± 0.5	24.2 ± 4.2	7.0 ± 0.9
120% PSF	29.4 ± 5.4	6.1 ± 0.8	30.9 ± 5.8	6.3 ± 1.0	20.4 ± 4.6	6.2 ± 0.7
stairmill Mean	35.0 ± 4.7	6.0 ± 0.1	36.2 ± 4.2	6.9 ± 0.4	24.7 ± 2.9	6.8 ± 0.3

### 2.4. Statistical analysis

A linear regression analysis was performed to determine the nature of the relationship between stride frequency during treadmill walking at PWS and stairmill climbing at PSF. The *p*-value and the overall percentage of variance accounted for by the regression (*r*^2^) was determined and reported. To investigate if there was an overall effect of task (stairmill climbing versus treadmill walking; 100% PSF on the stairmill and PWS on the treadmill) and joint angle (hip, knee and ankle) on the joint angle variability and the joint movement dynamics, a two-way ANOVA for repeated measures was applied. The dependent variables were meanSD, LyE and CoD and task and joint angle were the independent variables. To adjust for the number of one-way ANOVA tests completed in our previous study (15 in total) [16], the level of significance was corrected from 0.05 to 0.0034 using a Sidak-correction. In case any overall significant effect of the independent variables or the interaction (task × joint angle) met this adjusted level of significance, a Holm Sidak post hoc test was applied to investigate the between-task and between-joint differences. A similar statistical model was applied to investigate if there was an overall effect of the stepping frequency and joint angle (independent variables) on the joint angle variability and the joint movement dynamics (dependent variables). The results are depicted in box plots including the median value and the 5th, 25th, 75th and 95th percentile. The statistical analyses were performed in SigmaPlot (Systat Software, Inc. 2014, version 13.0, Germany).

## 3. Results

### 3.1. Stairmill climbing versus treadmill walking at PSF

The group mean, standard deviation and 95% confidence intervals of the stride frequencies during treadmill walking and stairmill climbing are presented in table 2. The stepping frequency during walking was approximately twice as high as during stairmill climbing at 100% PSF. The linear regression for the stride frequency during treadmill walking at PWS and stairmill climbing at 100% PSF revealed a non-significant (*p* = 0.518) relationship with *r*^2^ of 0.06.

**Table 2.** Group mean, SD, and upper and lower 95% confidence intervals for stepping frequency during stairmill climbing at 80, 90, 100, 110 and 120% PSF and treadmill walking at PWS.

	stairmill					
	80% PSF	90% PSF	100% PSF	110% PSF	120% PSF	walking PWS
mean ± s.d.	0.37 ± 0.08	0.41 ± 0.09	0.45 ± 0.09	0.49 ± 0.10	0.54 ± 0.10	0.94 ± 0.09
95% CI	0.31–0.42	0.35–0.47	0.39–0.51	0.42–0.55	0.47–0.61	0.88–1.00

**Figure 2.**
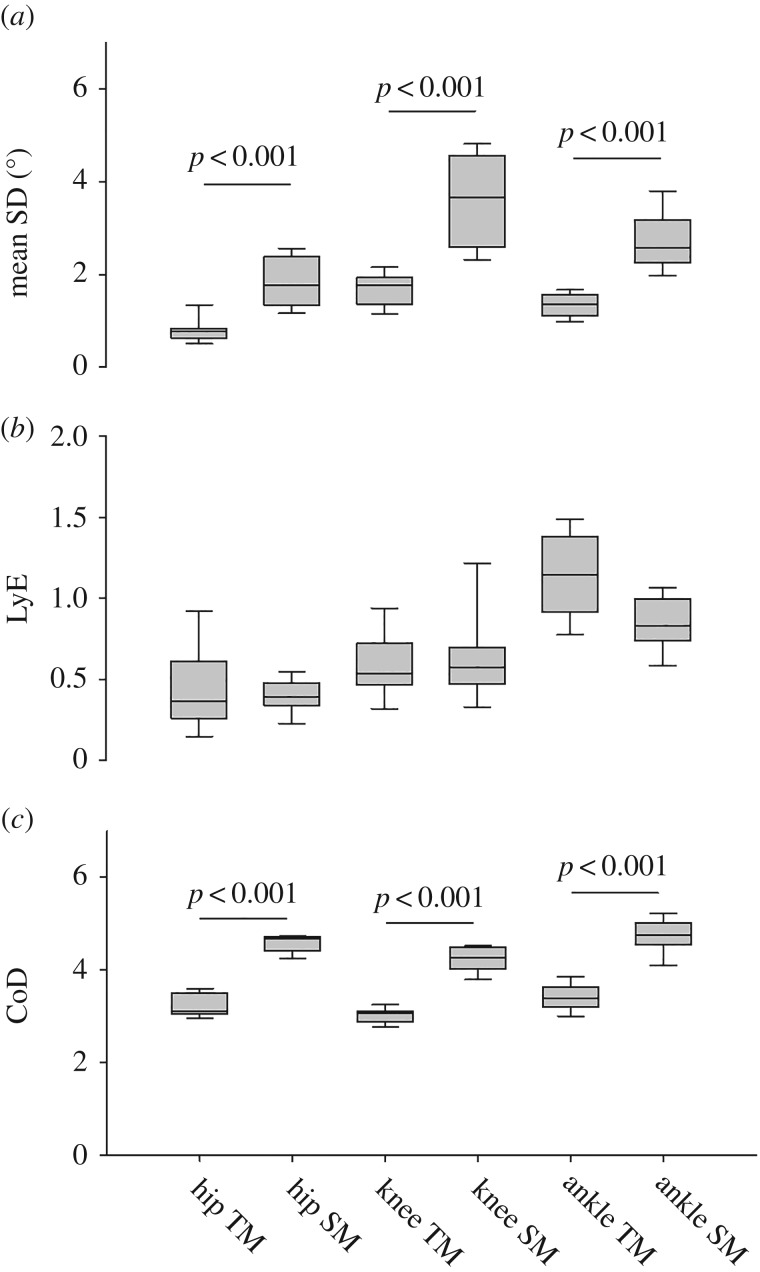
(*a*) Mean SD, (*b*) LyE and (*c*) CoD for the hip, knee and ankle joint angle during treadmill (TM) walking at PWS and stairmill (SM) walking at PSF.

There was a significant overall effect of both task (*F*_1,8_ = 45.6, *p* < 0.001) and joint (*F*_2,8_ = 68.2, *p* < 0.001) on the meanSD when comparing treadmill walking at PWS and stairmill climbing at 100% PSF (figure 2*a*). Post hoc tests revealed that the joint angle variability was significantly higher during stairmill climbing compared to treadmill walking (*p* < 0.001). Furthermore, the knee joint had significantly higher variability compared with the hip and the ankle joint (*p* < 0.001) and the ankle joint had significantly higher variability compared with the hip joint (*p* < 0.001, figure 2*a*).

There was a significant overall effect of joint (*F*_2,8_ = 96.5, *p* < 0.001) but not of task on the LyE (figure 2*b*). The post hoc tests revealed that the LyE of the hip joint angle was significantly lower compared with the more distal joint (*p* < 0.001 in all cases) and the LyE of the knee joint angle was significantly lower compared with the ankle joint angle (*p* < 0.001, figure 2*b*).

There was a significant overall effect of task (*F*_1,8_ = 180.1, *p* < 0.001) and joint (*F*_2,8_ = 58.5, *p* < 0.001) on the CoD (figure 2*c*). The post hoc tests revealed that the CoD during stairmill climbing was significantly higher compared with treadmill walking (*p* < 0.001) and that the knee joint angle CoD was significantly lower compared with both the hip and ankle joint CoD (*p* < 0.001 in both cases). Furthermore, the ankle joint angle CoD was significantly higher compared with the hip joint angle CoD (*p* < 0.001, figure 2*c*).

### 3.2. Effect of stepping frequency during stairmill climbing

**Figure 3.**
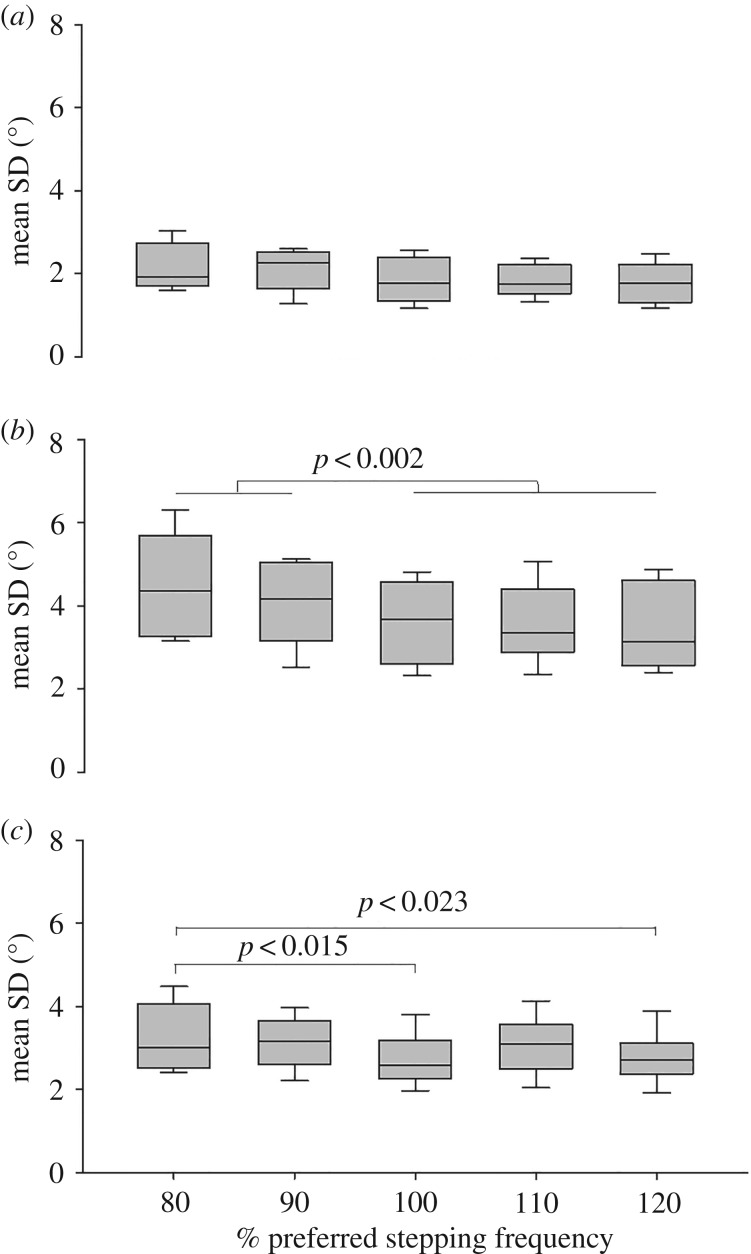
Mean SD of the (*a*) hip joint, (*b*) knee joint and (*c*) ankle joint angles during stairmill walking at 80, 90, 100, 110 and 120% of PSF.

There was a significant overall effect of both stepping frequency (*F*_1,8_ = 45.6, *p* < 0.001), joint (*F*_2,8_ = 9.4, *p* < 0.001) and the interaction (*F*_2,16_ = 3.7, *p* = 0.001) on the joint angle meanSD when comparing different stepping frequencies during stairmill climbing (figure 3). The post hoc tests revealed no differences in the hip joint angle variability with change in stepping frequency (figure 3*a*). The knee joint angle variability at the two lowest stepping frequencies was significantly higher compared with higher stepping frequencies (*p* < 0.002 in all cases, figure 3*b*). The ankle joint variability at 80% PSF was significantly higher than 100% PSF and 120% PSF (*p* < 0.021). Furthermore, the knee joint variability was in general significantly higher compared with the hip and ankle joint variability and the ankle joint variability was significantly higher than the hip joint variability (*p* ≤ 0.023 in all cases, figure 3*c*).

**Figure 4.**
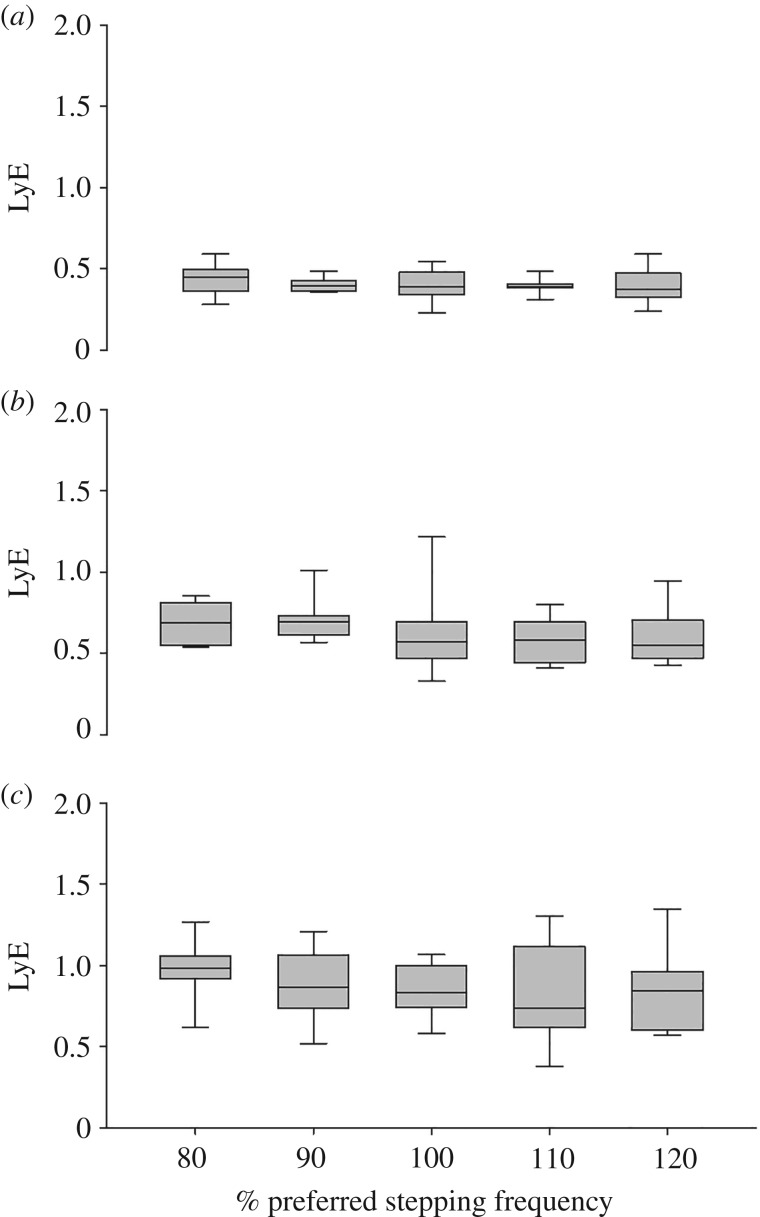
LyE of the (*a*) hip joint, (*b*) knee joint and (*c*) ankle joint angles during stairmill walking at 80, 90, 100, 110 and 120% of PSF.

There was a significant overall effect of joint (*F*_2,8_ = 42.4, *p* < 0.001) on the LyE (figure 4). There was no effect of stepping frequency or an interaction between joint and stepping frequency. The post hoc tests revealed that the LyE of the hip joint angle was significantly lower compared with the more distal joint (*p* < 0.001 in both cases) and the LyE of the knee joint angle was significantly lower compared with the ankle joint angle (*p* < 0.001).

**Figure 5.**
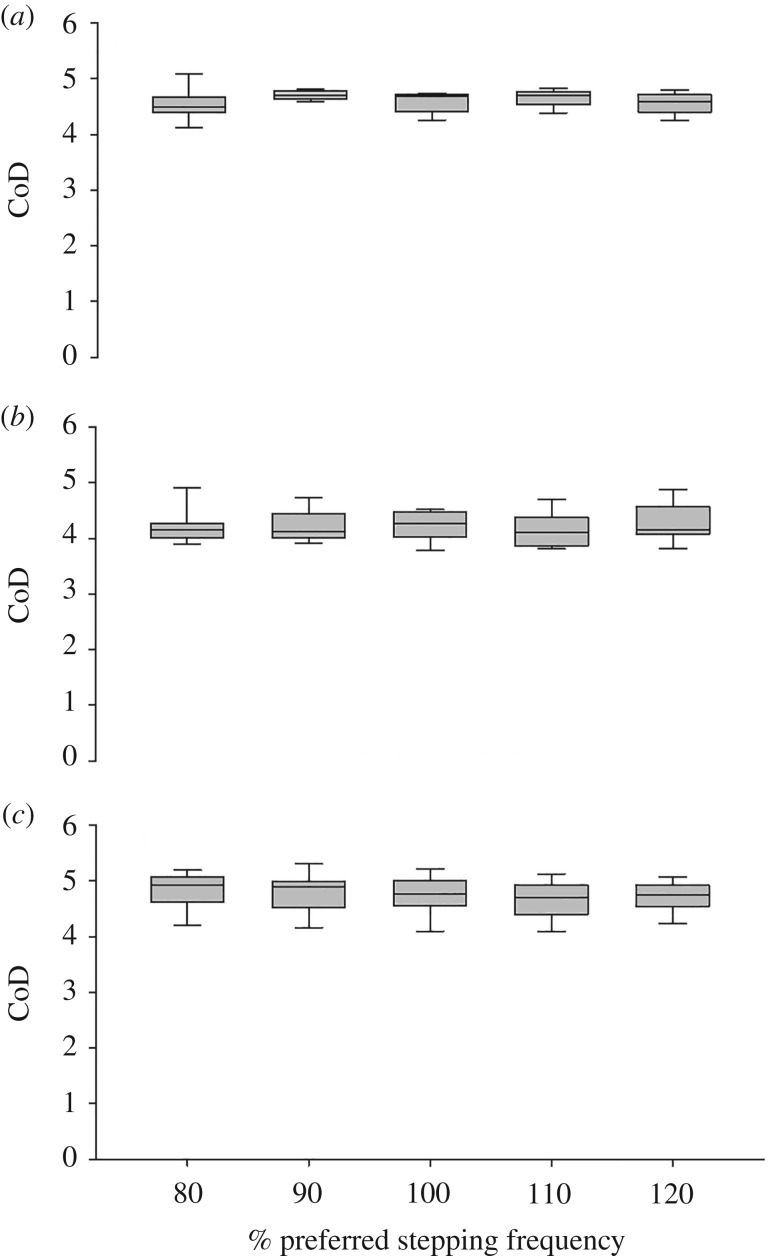
CoD of the (*a*) hip joint, (*b*) knee joint and (*c*) ankle joint angles during stairmill walking at 80, 90, 100, 110 and 120% of PSF.

There was a significant overall effect of joint (*F*_2,8_ = 32.4, *p* < 0.001) on the CoD (figure 5). There was no effect of stepping frequency or an interaction between joint and stepping frequency. The post hoc tests revealed that the CoD of the knee joint angle was significantly lower compared with the hip and ankle joint (*p* < 0.001 in both cases) and the CoD of the hip joint angle was significantly lower compared with the ankle joint angle (*p* = 0.046).

### 3.3. Effect of the number of included strides

The results of the additional analyses showed that the observed between-task and between-joint differences for both LyE and CoD were not affected by the number of included strides. The absolute values were affected by the number of included strides, especially the CoD, and comparisons with other studies using a different number of strides should be done with caution.

## 4. Discussion

Application of Dynamical Systems Theory to the motor control of human movement has previously suggested that optimal movement solutions are formed through the coordination of the multiple functional degrees of freedom within the human movement system while including the inherent interaction between environmental, biomechanical, biological and task-related constraints [14,15,32,33]. Accordingly, the existence of quadratic relationships between stepping frequency and walking speed on the one hand and the gait variability, on the other hand, suggests that an optimal movement solution should exist during walking at 100% PSF and PWS. The present study explored if this was also the case with another locomotor task, continuous stairmill climbing. Thus, the purpose of the present study was to investigate the joint angle dynamics and joint angle variability during continuous stairmill climbing at stepping frequencies above and below 100% PSF. It was hypothesized that the dynamics of the oscillatory leg movement would not differ between stairmill climbing and treadmill walking. Our results could not fully confirm this hypothesis. Although we did not observe any differences in the rate of trajectory divergence for the hip, knee and ankle joint angle between stairmill climbing at PSF and treadmill walking at PWS, we did observe significantly higher joint variability for all three joint angles during stairmill climbing at PSF compared to treadmill walking at PWS. Furthermore, greater joint dimensionality, quantified by CoD, was observed for stairmill climbing. Additionally, the present study investigated the effect of stepping frequency on joint variability and dynamics. It was hypothesized that joint variability would increase and that rate of trajectory divergence and joint dimensionality would increase during stairmill climbing at stepping frequencies above and below 100% PSF. This hypothesis was only partly confirmed for the knee and ankle joint angles where a higher variability was observed for stepping rates below 100% PSF.

While Dynamical System Theory seems to explain the movement dynamics during walking at PSF and PWS, the present study cannot support that continuous stairmill climbing, regardless of its apparent walking-like appearance, shares similar dynamics with walking. While the biological and biomechanical constraints (intrinsic physical and psychological limitations of the human system) are the same during stairmill climbing and treadmill walking, the environmental and task constraints are different. On the stairmill, the appearance of the stair-steps follows a rhythmic pattern to which the participant's movement solution needs to be adjusted. Furthermore, the stairmill climbing task requires foot-steps with a minimum of foot lift and a narrow range of step lengths. Changes in similar parameters have previously been shown to alter the joint dynamics during regular treadmill walking. Specifically, constraints introduced during treadmill walking in terms of a narrow and straight walking path and a constant speed has been observed to lead to lower joint movement variability compared with overground walking [34–36]. In contrast to these observations, the joint angle movements, in this study, were more variable during stairmill climbing suggesting that a higher number of different movement solutions were used to solve the task of stairmill climbing. We suggest two alternative explanations for these observations. One explanation could be that the interference of environmental and task constraints with the interacting dynamics of available degrees of freedom increased both the variability and dimensionality of the joint angle movements compared with treadmill walking. The rhythmic pattern of stair-steps forced an altered stride time interval pattern compared to the freely chosen pattern on the treadmill [16]. This can be supported by the observations of previous studies using auditory cues during treadmill walking. When healthy adults were asked to synchronize their footstep to rhythmic auditory cues given by a metronome, their stride time patterns changed from statistically persistent during unconstrained walking to anti-persistent [37]. Additionally, our group has previously observed that while auditory cues with random (white noise) or periodic (metronome) temporal structures reduced the statistical persistence in stride time intervals, auditory cues that had a mathematically chaotic structure did not change the temporal structure of stride time intervals [38,39]. These observations suggest a clear sensitivity in the stride time dynamics during walking to external stimulus which, based on our previous observation, also appears to be present during stairmill climbing [16]. Furthermore, the foot clearance of each stair-step puts a larger demand on the hip flexion muscles during the swing phase compared with that during treadmill walking which potentially alters the oscillatory motion of the leg [40]. This consequently modifies the variability and dimensionality of joint angle dynamics in comparison to treadmill walking.

Alternatively, the higher joint angle variability during stairmill climbing could be interpreted as an exploration of different potential movement solutions in the search for an optimal movement attractor to solve the task of stairmill climbing [41]. This interpretation is supported by the higher fractal dimensionality observed for all joint angles during stairmill climbing which indicate a less tight organization of the trajectories in state space and an increase in the degrees of freedom. Furthermore, this would match the later part of the Bernstein's motor learning theory [42], suggesting that while stairmill climbing was not a novel task for the subjects, ongoing skill improvement was taking place during stairmill climbing [41]. During this process, if the negotiation of each step is considered to be a new problem to solve for the central nervous system with limited feedback from previous step(s), a more adaptive movement strategy could be used, characterized by a high dimensionality. This is equally supported by the observations of our previous study, where stride time and stride speed intervals during stairmill climbing had an uncorrelated temporal pattern [16]. Additionally, the results of the rate of trajectory divergence indicate that the rate of changes between the different movement solutions does not differ between stairmill climbing and treadmill walking. Therefore, it is possible that while the lack of skill mastering of stairmill climbing increases the number of movement solutions, it has no implication for the rate of change between different solutions. Although both explanations are valid, we consider the former to be most probably due to the effortless stairmill climbing performed by the subjects. However, because the results of the present study cannot exclude either of the two explanations, future studies should reveal whether joint angle variability and dynamics during stairmill climbing are susceptible to alteration following training.

The change in stepping frequency during stairmill climbing affected the knee and ankle joint angle variability but did not alter the corresponding dynamics. Thus, higher variability was observed at low stepping frequencies indicating that a higher number of movement solutions were used for two distal joints. This is in agreement with previous observations during treadmill walking [11], suggesting a common mechanism determining the movement variability during walking and stairmill climbing and different mechanisms determining the joint angle dynamics.

The rhythmic muscle activation during cyclic movements (including walking, running, cycling and knee extension exercise) has been suggested to originate from a neural network referred to as the central pattern generator (CPG) [43,44]. A linear relationship between the PSF during walking and the PSF during running in healthy individuals has been suggested to indicate that the two gait modalities share a common neural drive from the CPG [45]. Furthermore, a high correlation (*r* = 0.94) has been observed between the freely chosen frequencies during knee extension exercise for each leg and fair correlation (*r* = 0.71) has been observed between the freely chosen pedaling frequencies during one-legged cycling for each leg [46]. The authors of the aforementioned studies suggested that these significant correlations could indicate that the CPGs of the two legs share a common frequency generator or that separated frequency generators of each legs are attuned via interneuronal connections [46]. Adopting a similar approach in the present study, where the PSF during stairmill climbing and the stepping frequency during walking at PWS were not significantly correlated, would suggest that control of the rhythmic muscle activation during these tasks does not share a common neural drive from the CPG. Our previous observations on continuous stairmill climbing suggest that each step is considered a new problem for the central nervous system to solve and minimal feedback from previous step(s) is used to form future steps [16]. Based on this, it could be speculated that the requirement of adaptive control of stepping motion alters the CPG's influence on the stepping frequency. Future studies should investigate this topic.

Finally, while the results of the present study can be interpreted from two often opposing and contradicting theoretical frameworks (Dynamical System Theory and central motor program/optimization theory), it is worth mentioning that the formulation of Dynamic Motor Primitives by Schaal and colleagues offers a third alternative which combines the two aforementioned frameworks [47]. It is however beyond the scope of this study to apply this approach to the results.

The protocol of the present study only consisted of walking at PWS and did not include stepping frequencies above and below. Thus, we rely on the assumption that our subjects would respond to changes in stepping frequency in the same manner as observed previously [4,5].

## 5. Conclusion

During continuous stairmill climbing, joint angles exhibited increased variability and altered dynamics characterized by increased dimensionality compared with treadmill walking at PWS. By contrast, the rate of trajectory divergence did not differ between the two tasks. Additionally, joint dynamics did not change with change in stepping frequency, suggesting that PSF during stairmill does not facilitate an optimal movement solution compared to higher or lower stepping frequencies. While quadratic relationships between walking speed or stepping frequency and gait variability exist during treadmill walking, the present study does not support that this is also the case with stairmill climbing. We suggest that the task constraints of fixed stair-step height, width and rhythmic appearance prevents the same optimized self-organization of degrees of freedom during stairmill climbing as observed during treadmill walking.

## Supplementary Material

Supplementary_material.docx

## Data accessibility

The electronic supplementary material including data and Matlab codes for this manuscript is available at: https://www.unomaha.edu/college-of-education/biomechanics-core-facility/research/index.php under Related Resources and Stairmill Project Supplementary Material.
