# Supplementary material for: Lower limb joint angle variability and dimensionality are different in stairmill climbing and treadmill walking
Source: R Soc Open Sci. 2018 Dec 12;5(12):180996. doi: 10.1098/rsos.180996 (PMC6304153; doi:10.1098/rsos.180996)
Supplement: Supplementary_material.docx [file rsos180996supp1.docx]

**SUPPLEMENTARY MATERIAL**

**Title:**

Lower limb joint angle variability and dimensionality are different in stairmill climbing and treadmill walking

**Authors:**

Raffalt, PC.^1,2^, Vallabhajosula, S.^3^, Renz J.J.^4^, Mukherjee, M.^4^, Stergiou, N.^4,5^

**Affiliations:**

^1^Julius Wolff Institute for Biomechanics and Musculoskeletal Regeneration, Charité – Universitätsmedizin Berlin, Berlin, Germany.

^2^Department of Biomedical Sciences, University of Copenhagen, Copenhagen, Denmark.

^3^ Department of Physical Therapy Education, School of Health Sciences, Elon University, Elon, NC, United States

^4^ Department of Biomechanics, College of Education, University of Nebraska at Omaha, Omaha, NE, United States

^5^ Department of Environmental Agricultural and Occupational Health, College of Public Health, University of Nebraska Medical Center, Omaha, NE, United States

**Corresponding Author:**

Nicholas Stergiou, PhD

Department of Biomechanics and Center for Research in Human Movement Variability University of Nebraska at Omaha

6160 University Drive

Omaha, NE 68182-0860, USA.

Email: [nstergiou@unomaha.edu](mailto:nstergiou@unomaha.edu)

Phone: 402-554-3247

# Purpose

The purpose of the present supplementary material was to investigate the effect of the number of included strides on the largest Lyapunov exponent (LyE) and correlation dimension (CoD) calculated from hip, knee and ankle joint angles.

# Methods

Each joint angle time series from the PSF stairmill trial was adjusted to include 10, 20, 30, 40 and 50 strides and each time series from the PWS treadmill trial was adjusted to included 10, 20, 30, 40, 50, 75, 100, 125 and 150 strides. LyE and CoD were calculated from each new time series using the same approach as described in the main study.

# Results

Figure S1 shows the LyE as a function of the number of included strides for both the PSF stairmill climbing and PSW treadmill walking trials. The LyE did not change substantially with increasing number of included strides during the two conditions for the hip and knee joint angles. There was a tendency to an increase in LyE with increasing number of included strides during treadmill walking but not during stairmill climbing for the ankle joint angle. Furthermore, changing the number of strides did not appear to alter the between-condition relationship in LyE, where no between-condition difference was observed.

Figure S2 shows the CoD as a function of the number of included strides for both the PSF stairmill climbing and PSW treadmill walking trials. The CoD had a curve linear increase with increasing number of included strides during the two conditions for all three joint angles. The higher CoD observed during stairmill climbing was present at all included number of strides for all three joints.

# Conclusion

The present supplementary material observed an effect of the number of included strides on the size of the CoD but not of the LyE calculated on the hip, knee and ankle joint angles. However, the between-condition relationship was not affected by the number of included strides for the two investigated parameters. In conclusion, the number of included strides in the present study did not appear to affect the overall outcome. Comparisons of results between the present study and other studies using a different number of strides should be done with caution.


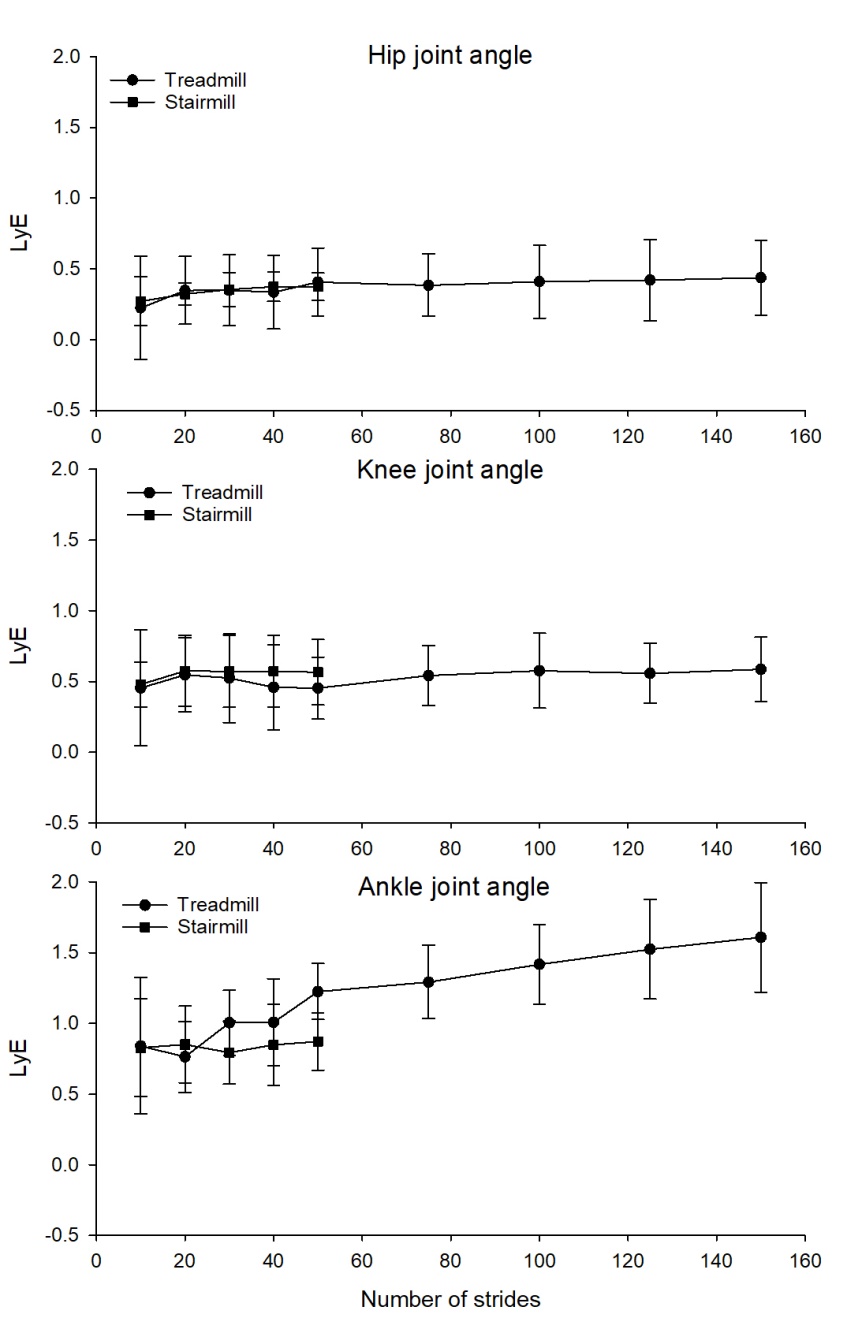


**Figure S1:**LyE of the hip, knee and ankle joint angle at different numbers of strides during stairmill climbing and treadmill walking.


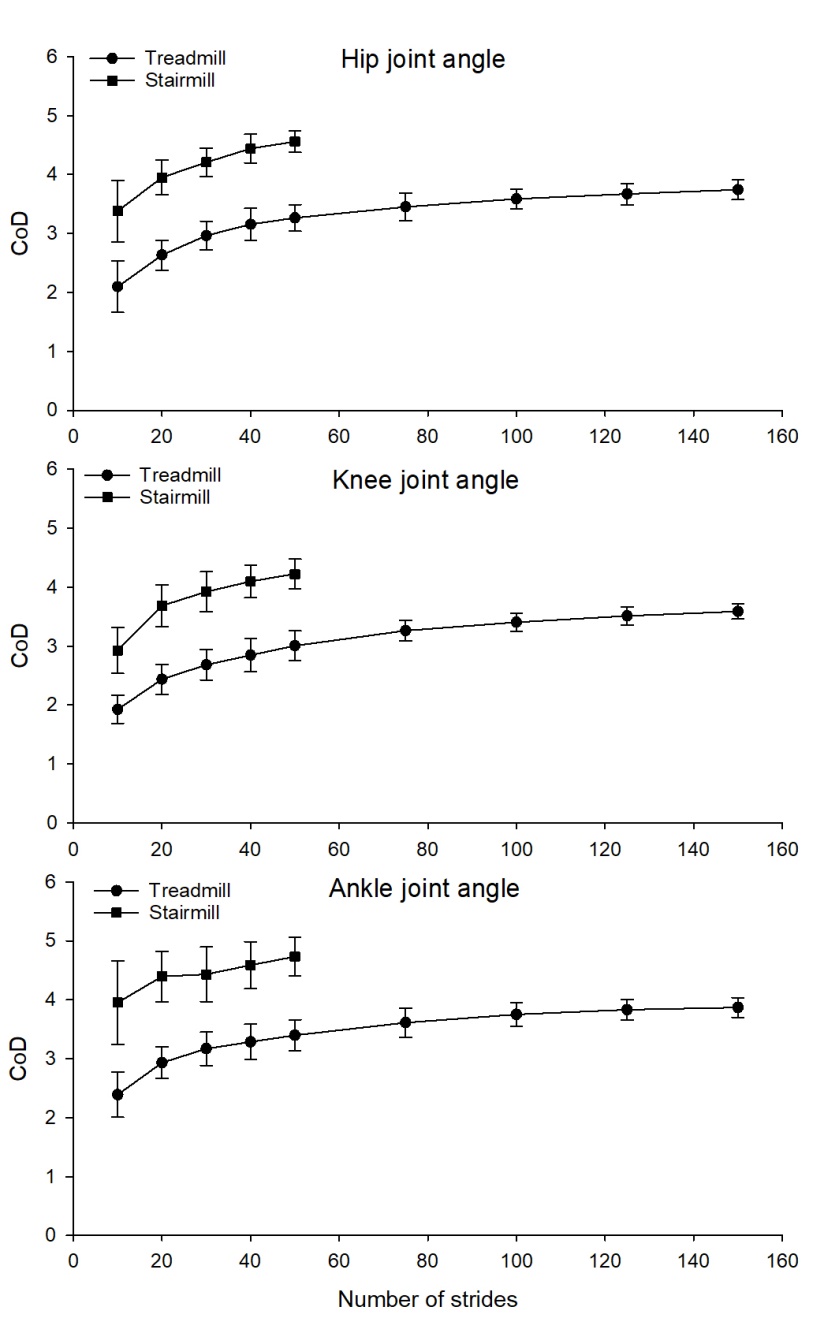


**Figure S2:**CoD of the hip, knee and ankle joint angle at different numbers of strides during stairmill climbing and treadmill walking.
